# The mediating role of social stress sensitivity on the relationship between hostile attribution bias and paranoia: An experience sampling study

**DOI:** 10.1192/j.eurpsy.2024.595

**Published:** 2024-08-27

**Authors:** A. K. C. Chau, S. H.-W. So

**Affiliations:** ^1^Department of Psychology; ^2^Institute of Health Equity, The Chinese University of Hong Kong, Hong Kong, Hong Kong

## Abstract

**Introduction:**

Heightened affective responses to daily life stressors, referred to as elevated affective reactivity to stress (or ‘stress sensitivity’), have been proposed as a putative mechanism of schizophrenia. Previous studies on stress sensitivity mainly used a case-control design; given that schizophrenia is heterogeneous its relationship with specific symptoms (e.g. paranoia) is yet to be addressed. In view of the continuum approach of understanding psychotic symptoms, the relationship between stress sensitivity (especially ‘social stress sensitivity’) and paranoia in the general population is important. Supported by emerging evidence of the relationship between hostile attribution bias (i.e. a tendency to interpret others’ actions as hostile and intentional) and paranoia, we hypothesized that social stress sensitivity mediates the relationship between hostile attribution bias and momentary experiences of paranoia.

**Objectives:**

Using experience sampling method, this study aimed to examine the association between social stress sensitivity, hostile attribution bias and momentary paranoia in non-clinical young adults. We also tested the role of social stress sensitivity as mediator of the relationship between hostile attribution bias and momentary paranoia.

**Methods:**

Consented participants free from any past and current psychiatric diagnoses (confirmed with the Structured Clinical Interview for DSM-IV Disorders) completed the measure of hostile attribution bias (i.e. abbreviated Ambiguous Intentions Hostility Questionnaire). Participants then filled in an ESM questionnaire measuring momentary levels of paranoia, social stress (i.e. pleasantness of and preference for being alone or with others) and negative affect on a mobile phone app repeatedly, ten times per day over six days. Social stress reactivity was calculated as the within-moment correlation between social stress and negative affect. The associations between social stress sensitivity, hostile attribution bias and momentary paranoia, and the mediating role of social stress sensitivity, were tested with multilevel modelling.

**Results:**

The final sample consisted of 131 participants (57.3% female, mean age= 20.36 (SD= 2.93)). The mean compliance rate was 71.9% (SD= 0.16). Social stress sensitivity was positively associated with momentary paranoia (B= 0.03, p= .002). Hostile attribution bias was associated with momentary paranoia (B= 0.41, p< .001), as well as social stress reactivity (B= 0.10, p= .003). The mediating effect from hostile attribution bias to momentary paranoia via social stress sensitivity was significant (ab= 0.05, 95% CI [0.03-0.07]).

**Conclusions:**

Social stress sensitivity was related to momentary paranoia, as well as hostile attribution bias. Our finding suggests social stress reactivity as a potential mechanism underlying the relationship between hostile attribution bias and paranoia.

**Disclosure of Interest:**

None Declared

